# Soaring seas, forest fires and deadly drought: climate change conspiracies and mental health

**DOI:** 10.1192/bjb.2021.7

**Published:** 2021-08

**Authors:** Alexander Jack, Reena Panchal

**Affiliations:** University of Wolverhampton, UK

**Keywords:** Psychotic disorders, depressive disorders, personality disorders, phenomenology, schizophrenia

## Abstract

There is scientific consensus that anthropogenic climate change is real and that it provides an existential threat to humanity and the planet. In this article, we focus on climate change conspiracy theories and the impact of such beliefs on mental health. We discuss the psychiatric disorders that might be relevant to conspiracy belief endorsement and we present the underlying psychological mechanisms. We note that there is little to no literature to associate beliefs about climate change with serious mental health conditions. However, we anticipate that such beliefs may manifest pathologically in psychiatric presentations as climate change becomes increasingly at the forefront of the global agenda.

There is a consensus among climate scientists that anthropogenic climate change is real, harmful to the environment and a threat to our futures.^1^ Despite this, climate change denial and scepticism are prominent in social and political discourse, with support from influential public figures.^2^ Conspiracy theories are bedfellows of climate denialism and scepticism and have been defined as ‘attempts to explain the ultimate causes of significant social and political events and circumstances with claims of secret plots by two or more powerful actors’.^3^ Climate change conspiracy theories are popular, with endorsement between 20%^4^ and 40%^5^ in the USA. In other Western countries, denial is less prevalent, though still present.^6^

Douglas & Sutton^2^ noted climate change conspiracy theories to follow four main themes: scientists are faking climate change (a) for political reasons or (b) to secure research funding, or climate change is a hoax to (c) enable the ‘green agenda’ and (d) promote nuclear power. Notably, each theme implicates ‘scientists’ in perverse and deceptive actions. It would appear that this is required to undermine the consensus position among experts.

## Why do people believe climate change conspiracy theories?

Douglas et al^7^ suggested that epistemic, existential and social motivations underpin conspiracy beliefs. Epistemic motivations relate to the extent to which individuals or groups hold knowledge of phenomena that affect their lives. Conspiracy beliefs are strengthened when events are significant and/or wide-reaching^8^ and mainstream explanations are simplistic or lacking emotional charge.^9^ Conspiracy beliefs can provide a sense of understanding in the face of contradiction and uncertainty, and may offer closure when mainstream narratives do not provide satisfactory explanations.^10^ Existential motivations relate to the experience of anxiety, threat or a perception of powerlessness in the face of danger. In such instances, conspiracy theories may provide safety.^11,12^ Finally, social motivations concern issues of power and hierarchy, with conspiracy theories more likely to emerge in those who have experienced oppression, victimisation or persecution.^13^ In such circumstances, groups can become insular and develop an ‘us versus them’ narrative.^14^ Safety is achieved through a narcissistic defence, whereby power is transferred from perceived ‘elites’ to the conspiracy belief holders.^15^ Of note, such groups are predominantly single and isolated ethnic-minority males with lower socioeconomic status and educational achievement.^16^

Holding one conspiracy belief increases the likelihood of believing another^17^ and individuals predisposed to general conspiracy thinking are more likely to deny climate change.^18^ An underlying tendency to prioritise counter-narrative explanations and to distrust institutions may be apparent. The notion of political socialisation is important (e.g.^13^); climate change denial is part of the conservative rhetoric and conspiracy talk that is common among world leaders such as Trump^19^ and Bolsonaro.^20^ Political polarisation of climate attitudes has been well-evidenced in the USA^21^ and Europe,^6^ among other regions. Climate change conspiracy theories might suggest allegiance to a particular conservative world view and be phenomenologically different from more emotionally charged beliefs such as the QAnon and COVID-19 conspiracy theories.

## Conspiracy theories, overvalued beliefs and delusions: challenges with definitions

Psychiatrists work with symptoms and diagnoses that are based on criteria set out by manuals such as DSM-5 and ICD-10. When criteria are met, a diagnosis can be made and a treatment plan devised. When thinking about conspiracy theories, the psychiatric terms ‘overvalued ideas’ and ‘delusions’ are challenged. Psychiatry defines ‘overvalued ideas’ as an erroneous response to an idea –‘an acceptable, comprehensible idea pursued by the patient beyond the bounds of reason. It is usually associated with abnormal personality’.^22^ The overvalued idea may be based on true evidence. The term was coined by Wernicke in 1906 and has further been explored by Jaspers and Fish, who made various suggestions of how to distinguish overvalued ideas from delusions.^23^ A delusion is defined as a ‘false, unshakeable idea or belief that is out of keeping with the patient's background; it is held with extraordinary conviction and subjective certainty’.^22^ Consequently, there can be a diagnostic dilemma as to which category climate change conspiracies – or any conspiracy theory beliefs – best align.

## The evidence supporting climate change conspiracy theories

Conspiracy theory beliefs are widespread (e.g.^24,25,26^). The internet is rife with ‘fake news’ and ‘misinformation’. Social media platforms, blogs and a whole range of other websites are dedicated to the mass propagation of ‘evidence-base-less’ theories that directly refute scientific findings.^27,28^ Bye Bye Blue Sky is one of many groups that promote and raise awareness of climate change conspiracy theories. For example, their mission statement describes ‘chemtrails’ and a government conspiracy to control the weather as ‘the toxic spraying of nano particulate metals which are further amplified by ionospheric heaters to steer, direct and control our weather for military purposes … we seek to apply the wealth of our knowledge, passion and talents to end the illegal spraying of our planet’ (byebyebluesky.com/). Alarmingly, the internet content and group demonstrations can be very convincing. Furthermore, some theorists have mastered the ability to navigate through such forums and use them to their advantage to disseminate their views to a large population.^29^

An interesting quality of conspiracy theories is that the counter-evidence is shunned by theorists who claim that the refutation of their ideas by powerful figures is further evidence of truth suppression. This introduces an ‘us versus them’ dynamic.^14^ It may also have a directly proportionate effect – the stronger the evidence against the conspiracy theory, the more vehemently the counter-narrative is held.^30^ This is further reinforced by computer algorithms that provide a feed of confirmatory evidence and omit counter-narratives from view.^31^ Curiously, it is evident that the scientific community's message on climate change does not have the same footing or far-reaching sustenance as the competing conspiracy theories.

Referring back to the traditional definitions of overvalued ideas and delusions, it seems that conspiracy theories about climate change could be categorised as either or neither. A diagnostic challenge is introduced when groups or ‘masses’ of believers share the same conspiracy theory and it almost has the constructs of a culture. Studies^4,5^ have found the existence of large populations that believe conspiracy theories, supporting the idea that such beliefs extend beyond the individual. This directly causes conflict with the definition of delusions. It emphasises the need for clinicians to consider the presence of additional psychopathology and/or functional impairment in order to make a diagnosis in an individual who is preoccupied with conspiracy beliefs. It could be postulated that this may divide psychiatrists. Some may consider that this group hold pathological beliefs, whereas others may frame the beliefs as in keeping with a ‘subculture’ and therefore not indicative of psychopathology.

## Climate change conspiracies and mental disorders

Although there is an abundance of literature on climate change conspiracy theories in terms of their nature and spread, there appears to be very little on how such theories have had a clinical effect on mental health. In this section, the link between climate change conspiracy theories and mental disorders is discussed.

### Personality disorders

Individuals with certain personality disorder diagnoses are likely to be more susceptible to preferring narratives engineered by conspiracy beliefs due to the nature of the definition in diagnostic manuals such as ICD-10. In particular, the cluster A personality disorders have the fitting profile. The description of paranoid personality disorder specifically makes reference to a ‘preoccupation with unsubstantiated conspiratorial explanations of events both immediate to the patient and in the world at large’. Additional traits include recurrent suspicions without justification, general suspiciousness and a pervasive tendency to distort experience by misinterpreting neutral actions of others as hostile or malicious. Persons with a diagnosis of paranoid personality disorder may also have a rather rigid world view and an assertive sense of personal rights, which may not be proportionate to actual situations.^32^ It is apparent how this profile might cross over with characteristics identified in those who endorse conspiracy theories (e.g.^7^).

The ideas characteristic of paranoid personality disorder are typically persecutory and self-referential. Individuals diagnosed with this disorder are likely to be socially withdrawn and perceive that they are unduly victimised.^33^ Imhoff & Lamberty^34^ noted similar characteristics in relation to subclinical paranoia. However, instant access to widespread networks, facilitated by the internet, allows the formation of clusters of like-minded individuals who also hold similar persecutory, self-referential ideas. There is now a mechanism by which such individuals can indulge pathologically in misinformation to bolster their false beliefs together and ‘connect’. Therefore, in this scenario, self-referential ideas become a collective experience.^35^ This could further add opposition to the mainstream narrative and have an impact on individual presentation.

The criteria for schizoid personality disorder include traits such as an ‘invariable preference for solitary activities’, ‘a lack of close friends or confiding relationships’, ‘poor acknowledgement of social norms and conventions’ and, importantly, ‘excessive preoccupation with fantasy and introspection’.^32^ A combination of such traits could underpin a tendency to believe conspiracy theories.

Schizotypy is also implicated in the conspiracy theory literature.^36^ Schizotypy is captured in DSM-5 as a personality disorder and categorised with schizophrenia in ICD-10. March & Springer^37^ explored whether the ‘odd beliefs’ and ‘magical thinking’ seen in schizotypy predicted belief in conspiracy theories and found a significant association between the two. The authors commented that the results indicated that individuals with ‘unusual patterns of thinking and cognitions’ and ‘interpersonal and affective’ deficits were more likely to hold conspiracy beliefs. There are indications that particular personality traits are risk factors for psychosis in an attenuated form.^38,39^

### Nihilistic and apocalyptic delusions in psychotic depression

Severe depressive disorders may have a psychotic component in which mood-congruent delusions are a feature. Nihilistic delusions, where the patient has abnormal conviction that they are dead, their organs are rotting or the world is dead around them, are not uncommon.^40^ There is a possibility that this belief could extend to an individual believing that they are personally responsible for climate change or – in extreme cases – the destruction of the world.

Another type of delusion referred to in the literature is ‘apocalyptic delusions’ or ‘end-of-the-world delusions’. The content of such delusions is thought to be influenced by contemporary culture and societal changes. Early content of such delusions included fears of the plague, famine and asteroids hitting the earth.^41^ Although these persist, the content has evolved in the present day, as would be expected, and includes despair over climate change. ‘Climate apocalypse’ and ‘climate dystopia’ְ are terms that encompass the idea that an apocalypse will occur as a result of climate change – severe weather changes, forest fires and a depletion of natural resources will render the earth uninhabitable and therefore bring about the inevitable impossibility of the survival of human life.^42^

There is a single published case study to describe this phenomenon. Wolf & Salo^43^ described a 17-year-old boy diagnosed with a depressive disorder, who developed a delusion that his consumption of water would lead to the deaths of millions of people, as water supplies would be depleted. This was associated with ‘visions’ of an apocalypse.

Overall, there is a lack of recorded clinical cases of severe depression related to climate change or climate change conspiracy theories. However, with heightened attention on and uncertainty about climate change in modern society, there may be an increase in manifestations of this in depressive disorders through the modes suggested.

### Psychotic disorders

There are controversial terms such as ‘mass delusion’, ‘climate alarmists’ and ‘greenhouse sceptics’, which refer to various groups of people who hold certain beliefs about climate change.^44^ There are conspiracy beliefs propagated by some ‘climate deniers’ to state that climate change is a hoax.^45^ Counter-conspiracy beliefs also exist, which propose that the impact of climate change is understated, data are suppressed and governments are purposefully minimising the accelerating impact on the earth to fit with their political agendas.^2^ In terms of psychiatric diagnosis there is no evidence to suggest that such beliefs have a delusional quality. The terms ‘mass delusion’ and ‘climate deniers’ do not have clinical connotations. Nonetheless, there is likely to be a minority with associated risk factors for psychiatric disorders within the groups who are prone to holding these beliefs with absolute conviction despite contrary scientific evidence. Such delusions may be considered part of an evolving clinical picture of a delusional disorder, or a psychotic disorder such as schizophrenia. Considering the nature of these disorders, if climate change delusions were present, they would be expected to have a bizarre quality; and it would not be unusual for extreme conspiracy theories to be the themes.

There is a small literature base – and accompanying anecdotal evidence – concerning the interplay between sociocultural events and delusional content. For example, Cannon & Kramer^46^ have noted that delusional content in the USA related to syphilis in the early 20th century, Nazis during Second World War, communists during the Cold War and technology in more recent years. The internet has become increasingly relevant to delusional content (e.g.^47,48,49^). Curious case studies also exist. For example, Caseiro & Queiros^50^ reported a case in which football was thematic, in the context of Portugal winning Euro 2016. Notably, psychosis is often triggered by real-world events and the nature of delusional content can reflect genuine concerns about the world, anxiety and existential threat.^51^

It is possible that concerns about climate change could exacerbate existing delusional beliefs, or extreme views could escalate above a delusional threshold. Consequently, such beliefs could become ‘diagnosable’ and meet criteria for a psychotic disorder.

## A psychological adjunct

There is an association between the cognitive and affective processes that underpin conspiracy beliefs and those evidenced in delusional thinking. For example, the jumping-to-conclusions bias has been observed in psychotic-like thinking.^52^ This bias is associated with the overly rapid appraisal of stimuli to form a conclusion and has recently been evidenced in a sample of conspiracy theory believers.^53^ Poorer analytical thinking is also implicated,^54^ and cognitive distortions noted in depression could also be relevant.^35^ Similarly, historical victimisation and a schematic view of the world as dangerous are risk factors for psychosis^55^ and can provide a framework through which anomalous information is perceived. Individuals with schizotypal and paranoid personality disorder diagnoses are also likely to have experienced danger in their early lives^56,57^ and such threatening experiences are also precipitants to conspiracy beliefs.^11,13^ Distortions in human information processing are commonplace, adaptive and dimensional, with evidence to suggest that some individuals perceive ‘true’ information, whereas others omit, deny or delude as their environmental niche requires.^58^ Events that elicit threat responses are particularly relevant. Additionally, the demographic profiles of those who endorse conspiracies^16^ are similar to those found within psychosis cohorts.^59^

Many theorists and clinicians support the concept of dimensional psychosis with phenomenological continuity.^60,61^ Subclinical delusional thinking has been associated with conspiracy beliefs,^62^ as has paranoia.^34,63^ Conspiracy theorists may not be delusional or paranoid. However, it is plausible that they exhibit similar genetic, psychological and/or social characteristics to those who are vulnerable to psychosis. The proneness–persistence–impairment model^61^ and extended psychosis phenotype^64^ are helpful frameworks for exploring the relationship. For many, a belief in climate change conspiracies could simply concern loyalty to conservative values.^13^ However, some may have an underlying predisposition to psychosis, with a tendency towards conspiracy thinking. Potential migration towards clinical thresholds could occur in response to greater perceived threat from significant events and exposure to, and preoccupation with, conspiracies. Individuals with diagnoses of paranoid or schizotypal personality disorder may fall into this position. The notion of the extended phenotype could explain why individuals who hold one conspiracy belief are more prone to believing others (e.g.^17^); this has also been called ‘conspiracist ideation’.^65,66^

## Forecast

There is evidence to suggest that historical events have informed delusional content^46,49^ and there are some indications that the COVID-19 pandemic has had a recent effect.^67^ Delusions can be triggered by real-world events and the content can reflect genuine concerns about the world, anxiety and existential threat.^51^ Notably, the conditions in respect to the above phenomena were opportune for the development of conspiracy theories. That is, these events generated real threat to individuals and communities (e.g.^7^).

It can be hypothesised that the global reluctance/opposition to impactful climate policy change is actually protective with regard to the pervasive development of conspiracy belief psychopathology. That is, conspiracy theories emerge when a way of life is threatened. At present, climate policy has not had a tangible impact on freedoms, rights or lifestyle. Nonetheless, we forecast that this will likely change in the coming decades (e.g. as governments fall in line with the Paris Agreement). To our knowledge, there is only a single case study^43^ to describe the interplay between climate change conspiracy beliefs and severe psychopathology. It is hypothesised that climate change has not yet become a common feature of delusional beliefs.

For those who have a predisposition to psychopathology, such changes may trigger migration towards clinical disorder. It is possible that climate change and associated conspiracy beliefs may affect mental health in the following ways: (a) dramatic societal changes (e.g. energy conservation policies, restrictions on existing freedoms) might precipitate an increase in climate change conspiracy theories, and individuals predisposed to conspiracy thinking (including those with underlying paranoid, schizoid and schizotypal traits) might migrate to delusion; (b) the increasing presence of climate change discourse in public conversations could lead to such content appearing in the content of pre-existing delusional belief systems; and (c) concerns and guilt about climate change could lead to presentations of depressive psychosis with nihilistic and/or climate-related apocalyptic delusions.

## Concluding remarks

As climate change becomes more present in public consciousness, conspiracy theories are likely to become increasingly prominent and to manifest in the presentation of several mental disorders. This article has hypothesised that there may be a potential commonality between pervasive conspiracy thinking and mental disorders, particularly psychosis and certain personality disorders. However, it is proposed that clinicians approach individuals who hold conspiracy beliefs with diagnostic caution, given that conspiracy beliefs are widely held within the general population. Careful assessment is needed to identify those who are psychologically vulnerable to developing mental health complications due to exposure to conspiracy beliefs.

Further research is recommended to investigate whether a proportion of climate change conspiracy theory advocates do have underlying psychological risk factors for the development of concerning psychopathology; and also how such theories have featured in mental disorders, particularly as symptoms of psychosis or personality disorder.

## About the authors

**Alexander Jack** is a senior forensic psychologist in the Department of Psychology, Reaside Clinic, Birmingham and Solihull Mental Health Foundation Trust, Birmingham, UK. **Reena Panchal** is a Year 5 Specialty Trainee (ST5) in forensic psychiatry in the Department of Psychiatry, Reaside Clinic, Birmingham and Solihull Mental Health Foundation Trust, Birmingham, UK.
